# Adaptation and Psychometric Evaluation of the Chinese Counseling Competencies Scale-Revised

**DOI:** 10.3389/fpsyg.2021.688539

**Published:** 2021-06-21

**Authors:** Wei Xia, William Ho Cheung Li, Tingna Liang, Yuanhui Luo, Laurie Long Kwan Ho, Ankie Tan Cheung, Peige Song

**Affiliations:** ^1^School of Nursing, Sun Yat-Sen University, Guangzhou, China; ^2^School of Nursing, The University of Hong Kong, Hong Kong, China; ^3^School of Public Health, Zhejiang University School of Medicine, Hangzhou, China; ^4^Centre for Global Health Research, Usher Institute of Population Health Sciences and Informatics, The University of Edinburgh, Edinburgh, United Kingdom

**Keywords:** psychometric evaluation, counseling competencies, addition counseling, counseling education, Chinese

## Abstract

**Objectives:** This study conducted a linguistic and psychometric evaluation of the Chinese Counseling Competencies Scale-Revised (CCS-R).

**Methods:** The Chinese CCS-R was created from the original English version using a standard forward-backward translation process. The psychometric properties of the Chinese CCS-R were examined in a cohort of 208 counselors-in-training by two independent raters. Fifty-three counselors-in-training were asked to undergo another counseling performance evaluation for the test-retest. The confirmatory factor analysis (CFA) was conducted for the Chinese CCS-R, followed by internal consistency, test-retest reliability, inter-rater reliability, convergent validity, and concurrent validity.

**Results:** The results of the CFA supported the factorial validity of the Chinese CCS-R, with adequate construct replicability. The scale had a McDonald's omega of 0.876, and intraclass correlation coefficients of 0.63 and 0.90 for test-retest reliability and inter-rater reliability, respectively. Significantly positive correlations were observed between the Chinese CCS-R score and scores of performance checklist (Pearson's γ = 0.781), indicating a large convergent validity, and knowledge on drug abuse (Pearson's γ = 0.833), indicating a moderate concurrent validity.

**Conclusion:** The results support that the Chinese CCS-R is a valid and reliable measure of the counseling competencies.

**Practice implication:** The CCS-R provides trainers with a reliable tool to evaluate counseling students' competencies and to facilitate discussions with trainees about their areas for growth.

## Introduction

Counseling has been proven to effectively promote many aspects of mental, psychological, and behavioral health (Bower et al., 2003; Lancaster and Stead, 2005; McFadden et al., 2019). Counseling is provided to individuals with problems in mental, psychological, or behavioral areas by counselors who are considered to be functioning well in these areas, relative to these individuals (Gladding, 2004). In their work, counselors draw on various principles of mental health, psychology or human development to implement cognitive, affective, behavioral or systemic interventions that address wellness, personal growth, career development, and pathology (Gladding, 2004). Notably, the effectiveness of counseling services depends on the quality and competence of the counselor (Agarwal et al., 2019).

To become a competent counselor, an individual must develop effective counseling skills and demonstrate a professional disposition and behavior (Hatcher and Lassiter, 2007; Fouad et al., 2009). Clinical counseling supervisors facilitate their supervisees' development and evaluate their mastery of the knowledge, skills, and professional disposition required of competent professional counselors [Council for Accreditation of Counseling and Related Educational Programs (CACREP), (2016)]. Clinical counseling supervisors also serve as gatekeepers to the profession and deny entry to those who do not achieve the necessary competencies [Association for Counselor Education and Supervision (ACES), 2011; Bernard and Goodsource, 2013; American Counseling Association (ACA), 2014]. Through evaluations, supervisors can evaluate their counseling students' skills and discuss their strengths and areas for growth, which ultimately enhance the practices and the services provided to clients (Lambie and Swank, 2016). Therefore, counseling educators and supervisors require tools with which to evaluate students' counseling skills, dispositions, and behaviors (Swank and Lambie, 2012; Swank, 2014).

Although clinical supervisors are expected to perform the above-described developmental and remedial evaluations of their supervisees' counseling competencies, few specific guidelines are available to direct the evaluation process (Hensley et al., 2003). Specifically, no consensus has been reached regarding the standardized evaluation criteria for determining a minimum level of counseling competency, and few tested assessments are available to measure supervisees' counseling competencies. This situation fosters subjectivity in supervisory assessments and potential remediation (McAdams and Foster, 2007; Karpenko and Gidycz, 2012). Recently, the Counseling Competencies Scale (CCS) was developed for this purpose, and empirical studies have indicated that this assessment instrument could comprehensively measure students' development of counseling competencies (counseling skills, disposition, and behavior) (Swank et al., 2012; Lambie and Swank, 2016; Lambie et al., 2018).

The CCS was initially developed by the Council for Accreditation of Counseling and Related Educational Programs (CACREP) (Swank et al., 2012). A refined version, the Counseling Competencies Scale-Revised (CCS-R), was subsequently promoted and validated (Lambie and Swank, 2016; Lambie et al., 2018). The CCS-R addresses two domains: (1) counseling skills (12 items) and (2) professional disposition and behavior (11 items). Supervisors can rate their supervisees' levels of counseling competency pertaining to the items by using a rubric of five response categories including harmful, below expectations, near expectations, meets expectations, and exceeds expectations. Previous studies have demonstrated the convergent validity of the CCS-R through significant associations with counseling Skills, Dispositions, and Behaviors (Swank et al., 2012; Lambie and Swank, 2016). The results of validation analyses revealed strong internal reliability (Cronbach's α = 0.96 for all items, Cronbach's α = 0.94 the for domain of counseling skills, Cronbach's α = 0.96 for the domain of professional disposition and behavior) and excellent inter-rater reliability (intraclass correlation coefficient [ICC] = 0.84) (Lambie et al., 2018), which supported the use of the CCS-R to measure the counseling competencies of trainees.

China is one of the most populous countries in the world. Currently, many Chinese citizens are experiencing increased behavioral, mental, and psychological distress in response to intensified social competition and rapid social change (Zhang, 2018). However, counseling training and services remain rare in China, and no counseling competency evaluation is currently available (Zhao, 2014; Ng et al., 2017). The introduction of standard tools for counseling competency evaluation may help to increase awareness about counseling and promote both quality training and best practices for the counseling services provided to Chinese clients. However, the CCS-R has not been adapted culturally or linguistically for the Chinese population. Therefore, this study aimed to adapt the CCS-R culturally for a Chinese population and to validate the psychometric indexes of the translated CCS-R in a cohort of Chinese counselors-in-training in Hong Kong.

### Conceptual Framework for Validation

According to the 2016 CACREP Standards (Council for Accreditation of Counseling and Related Educational Programs (CACREP), (2016)). the requirement for addiction counseling, the entry-level counselor students who are preparing to specialize as addiction counselors are expected to possess the knowledge and skills in the context of addiction counseling, treatment, and prevention programs, as well as in a more broad mental health counseling context (Council for Accreditation of Counseling and Related Educational Programs (CACREP), (2016)). Similarly, studies have shown that a counselor's knowledge of abuse is a major predictor of the quality of the counseling service and the treatment outcomes for substance abusers [Hensley et al., 2003; Association for Counselor Education and Supervision (ACES), 2011]. Inadequate knowledge may lead to negative consequences of addiction management (Hensley et al., 2003), therefore, the gatekeepers have to deny entry of those who do not master the necessary knowledge on drug abuse as addiction counselors. In addition, the counseling process is a planned, structured dialogue between a counselor and a client. The completion of the counseling procedure reflects that the counselor could basically use consulting skills, build relationships with clients, and respond to the communication (Carroll-Alfano, 2019). As stated above, the knowledge of drug abuse and the completion of counseling procedures were used as validation indexes of the CCS-R.

## Methods and Materials

**Figure 1:**
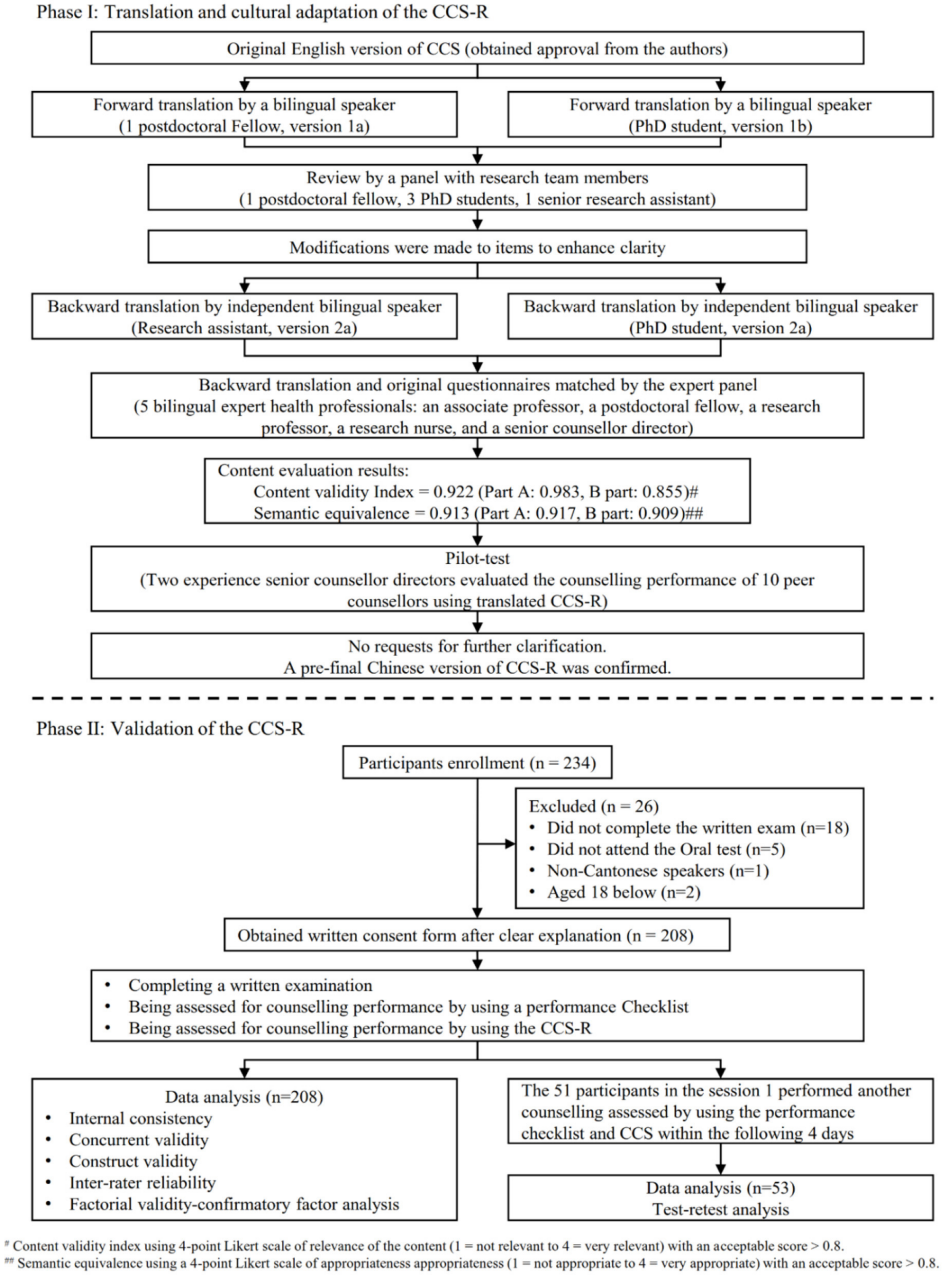
Flowchart of materials and method for the translation and validation of the CCS-R.

This descriptive study to determine the psychometric properties of the Chinese CCS-R was conducted in association with the Medical Peer Addiction Counseling (MedPAC) Quitline Service in Hong Kong. An expert panel of five bilingual experts from the fields of behavioral intervention, psychology, addiction intervention, and clinical counseling was created. We obtained the standard manual of the CCS-R and permission to translate the scale into the Chinese language from the authors and the CACREP. This study received ethical approval from the Institutional Review Board of the University of Hong Kong/Hospital Authority Hong Kong West Cluster (UW 20-395), and has been registered with ClincalTrial.gov (NCT04547517). This study was conducted according to the two major phases recommended in the Guidelines for Establishing Culture Equivalency of Instruments (Ohrbach et al., 2013): phase I, translation and cultural adaptation, and phase II, translation validation and documentation. A flowchart of the process is presented in Figure 1.

### Phase I: Translation and Cultural Adaptation

The original English version of the CCS-R was first translated into Chinese by a postdoctoral fellow who was knowledgeable about the scale content and a PhD student who was unfamiliar with the scale content to minimize bias. The two translated versions were synthesized into a single version, and discrepancies were discussed by the research team. After a review and modification by the expert panel, the Chinese version was back-translated into English by two independent bilingual translators without prior knowledge of the scale content. The expert panel reviewed and compared the backward translation with the original scale with the understanding that the translation process aimed to maintain conceptual rather than literal meaning (Wynd et al., 2003). Minor modifications of a few items were then made to enhance clarity, following the recommendations of the expert panel. Subsequently, the expert panel independently rated the content validity of the translated CCS-R on a 4-point scale (1 = not relevant to 4 = very relevant) and the semantic equivalence on a 4-point Likert scale of appropriateness (1 = not appropriate to 4 = very appropriate) (Norwood, 2000). The translated scale received a content validity index (CVI) of 0.922 (Part A:0.983, Part B:0.855; acceptable score >0.80, possible range: 0–1) and a semantic equivalence score of 0.913 (Part A:0.917, Part B:0.909; acceptable score >0.80, possible range: 0–1) (Supplementary Table 1).

Finally, the translated CCS-R was evaluated in a pilot test with a convenience sample of 10 counselors employed by a Youth Quitline, which provided telephonic smoking-cessation services. Written consent was obtained from the 10 counselors, who were then evaluated during the provision of telephonic counseling by two independent experienced senior counseling directors using the translated CCS-R. No requests for further clarification were made during the pilot test. A pre-final Chinese version of the CCS-R was confirmed and prepared for further evaluation.

### Phase II: Translation Validation and Documentation

#### Participants

The study participants included counselors-in-training and raters. Students enrolled in the MedPAC training course were approached to participate as counselors-in-training in the study. They were deemed eligible if they (1) had completed the training courses led by the MedPAC research team, (2) were aged 18 years or above, and (3) could speak Cantonese and read traditional Chinese. Participants were excluded if they failed to provide written consent and did not participate in an written test of counseling related test and oral test of counseling performance. According to the guideline that specified a respondent-to-item ratio of 5:1 (Tsang et al., 2017), a sample of at least 120 participants was required to cover the 23 CCS-R items evaluated in this study.

Of the 234 counselor-in-training approached for the study, 26 were excluded because they refused to participate (*n* = 3), did not complete the oral examination (*n* = 16), were not Cantonese speakers (*n* = 1) or were younger than 18 years (*n* = 6). Finally, 208 participants were included in this study.

The raters were four senior counseling supervisors and four senior counselors. The senior counseling supervisors held Master's or higher degrees and had at least 2 years of experience in counseling training and supervision. The senior counselors held Master's or higher degrees and had at least 2 years of counseling experience. All of the raters were female.

#### Measures

##### Counseling Competency Scale-Revised (CCS-R)

The 23-item CCS-R measures counseling competencies within two domains: (A) counseling skills and therapeutic conditions (12 items) and (B) counseling disposition and behavior (11 items) (Lambie et al., 2018). Each item is scored using five supervisor-rater evaluation response categories: (a) harmful, 1 point; (b) below expectations, 2 points; (c) near expectations, 3 points; (d) meets expectations, 4 points; and (e) exceeds expectations, 5 points. The scores of the items in each domain are then summed to yield two domain scores with possible ranges of 12–60 points and 11–55 points, respectively. A higher score indicates a better performance of counseling competencies.

##### Knowledge of the Drug Abuse

The knowledge of drug abuse was rated on a five-point scale with 10 items. All items were scored with 1 indicating “strongly disagree,” 2 indicating “disagree,” 3 indicating “really can't say,” 4 indicating “agree,” and 5 indicating “strongly agree,” excepting the item 6 scored oppositely. The scores of all items were summed with a range of 10–50 points. The higher scores indicated a better mastering of knowledge of drug abuse. The reliability of the Chinses scale has been empirically examined with a Cronbach's alpha of 0.88 (Beat Drugs Fund, 2021).

##### Performance Checklist for the Oral Test

The performance checklist was developed based on the “5A” approach (ask, advise, assess, assist, and arrange) following the recommendations of current guidelines for cessation counseling by the Youth Quitline research team (Fiore et al., 2008), and was validated in a previous study (Li et al., 2017). The checklist includes 21 items divided into five domains: ask, advice, assess, assist, and arrange. Each item is scored on a 3-point scale: 0 = not attempted, 1 = attempted but not satisfactory and 2 = satisfactory. The full performance checklist scores range from 0 to 42 points, with higher scores indicating a more comprehensive counseling performance.

### Procedure

Prospective counselors who attended the counseling training provided by MedPAC were screened and invited to participate in this study. After receiving an explanation of the study purpose and procedure, eligible counselors-in-training who agreed to participate were required to provide signed written consent. All of the participants were asked to complete a demographic form that included their age, gender, enrolled curriculum, and educational level at the time of training course registration. After completing the training course, all of the participants were invited to participate in a written evaluation of knowledge on drug abuse. Within 5 days, the participants then participated in an oral test rated by two independent counseling supervisors who used the CCS-R and performance checklist to evaluate the telephonic counseling provided to simulated drug abusers using a standardized scenario. Participants who completed this evaluation during the first oral test session were asked to undergo an additional counseling evaluation within the following 4 days (test-retest). After the oral test, feedback from the raters was collected and record. All of the procedures were conducted in spoken Cantonese and traditional written Chinese.

### Data Analysis

The participants' demographic characteristics and performance scores are summarized using descriptive statistics (e.g., means, standard deviations, frequencies, and percentages). All of the statistical tests were two-sided, and a *p* ≤ 0.05 was considered to indicate statistical significance.

Factorial validity was evaluated using a confirmatory factor analysis (CFA). The Akaike Information Criterion (AIC), Tucker–Lewis index (TLI), root mean square error of approximation (RMSEA), comparative fit index (CFI), goodness-of-fit (GFI), and standardized root mean square residual (SRMR) were used to evaluate the goodness of fit of the factor analysis models. The AIC is a criterion for the goodness of fit, with the lowest AIC indicating a preferable model. Cut-off values of ≥0.95, ≤ 0.06, ≥0.95, ≥0.90, and ≤ 0.08 were used for the TLI, RMSEA, CFI, GEI, and SRMR, respectively (Brown, 2006; Mvududu and Sink, 2013; Nunnally, 1994). The diagonal weighted least squares (DWLS) estimator was considered and used for the ordinal variables in the CCS-R. Values of 0.32, 0.45, 0.55, 0.63, and 0.71 indicated poor, fair, good, very good, and excellent factor loading, respectively (Swank et al., 2012). Items with factor loadings of <0.32 were removed (Gorusch, 1983). Next, initial one-factor and the original two-scale model analyses were performed (Jackson et al., 1993) using parameters based on the theoretical structure of the instrument and the modification indexes of AMOS. Then, the one-factor and two-factor models were modified and the analyses were re-performed. Finally, the one-factor and two-factor models were modified and the analyses were re-performed.

Further psychometric evaluation of the CSS-R was made using a bifactor model (Dueber, 2017). Internal reliability was assessed using coefficient omega, which is similar to Cronbach's α but overcoming the strong assumptions of unidimensionality and equal factor loadings of the latter. An omega value of 0.70 or above is recommended to demonstrate a reliable total score (Gu et al., 2017). Moreover, an omega hierarchical (omega *H*) value was obtained to assess the percentage of variation attributable to a single general factor. An omega *H* of at least 0.8 has been suggested to indicate reasonable unidimensionality (Rodriguez et al., 2016). In addition, factor score determinacy and construct replicability were evaluated by factor determinacy (FD) and *H* index values, respectively. An FD >0.9 indicates adequate factor determinacy, and an *H*-value >0.8 indicates adequate construct replicability (Rodriguez et al., 2016). A two-way mixed-consistency measures intraclass correlation coefficient (ICC) and 95% confidence interval were used to assess the test-retest reliability and inter-rater reliability (IRR). ICC values of 0.40–0.70 and >0.70 indicated acceptable and good test-retest reliability, respectively (Shultz et al., 2020). IRR values of 0.60–0.74 and ≥0.75 indicated moderate and high reliability, respectively (Cicchetti, 1994).

Studies have shown that a counselor's knowledge of health issue undergoing the counseling is major predictors of the quality of the counseling service [Hensley et al., 2003; Association for Counselor Education and Supervision (ACES), 2011]. The convergent validity was assessed to examine the extent to which the CCS-R was related to the knowledge of the drug abuse. The concurrent validity was assessed between the CCS-R and the performance checklist. Pearson's correlation coefficient (γ) was used to measure convergent and concurrent validity, and values of 0.30 and 0.50 were considered to indicate moderate and large correlations, respectively (Tabachnick and Fidell, 2013).

## Results

Of the 234 counselor-in-training approached for the study, 26 were excluded because they refused to participate (*n* = 3), did not complete the oral examination (*n* = 16), were not Cantonese speakers (*n* = 1), or were younger than 18 years (*n* = 6). Finally, 208 participants were included in the analysis. The participants' demographic characteristics are presented in Table 1. The mean age was 21.2 (SD = 3.56) years. The majority of the participants were aged 18–25 years (*n* = 185, 88.9%), were female (*n* = 144, 69.2%) and were undergraduate students (*n* = 167, 80.3%). The largest proportion of participants were enrolled in the curriculum of medicine (*n* = 73, 35.1%), and social science (*n* = 64, 30.8%). The mean scores of knowledge on drug abuse and performance checklist were 44.0 (SD = 4.14) and 35.6 (SD = 2.88), respectively. Fifty-three participants who were rated (CCS-R and performance checklist) on the first day completed a second rating within 4 days (Figure 1).

**Table 1:** Participants' demographic information and counseling skill performance.

	**Range**	***N* (%)/mean (SD)**
		**(*n* = 208)**
**Age**	18–40	21.2 (3.56)
18–25		185 (88.9)
26 or above		23 (11.1)
**Sex**		
Female		144 (69.2)
Male		64 (30.8)
**Curriculum enrolled**
Medicine		73 (35.1)
Social science		64 (30.8)
Science		30 (14.4)
Arts		21 (10.1)
Others		20 (9.6)
**Education level**
Undergraduate		167 (80.3)
Master		41 (19.7)
Score of knowledge on drug abuse	32–50	44.0 (4.14)
Performance checklist	21–42	35.6 (2.88)
Counseling competencies scale-revised	60–107	86.9 (7.86)
Counseling skills and therapeutic conditions	28–58	45.0 (6.17)
Counseling dispositions and behaviors	30–50	41.9 (2.87)

### Factor Validity

**Figure 2:**
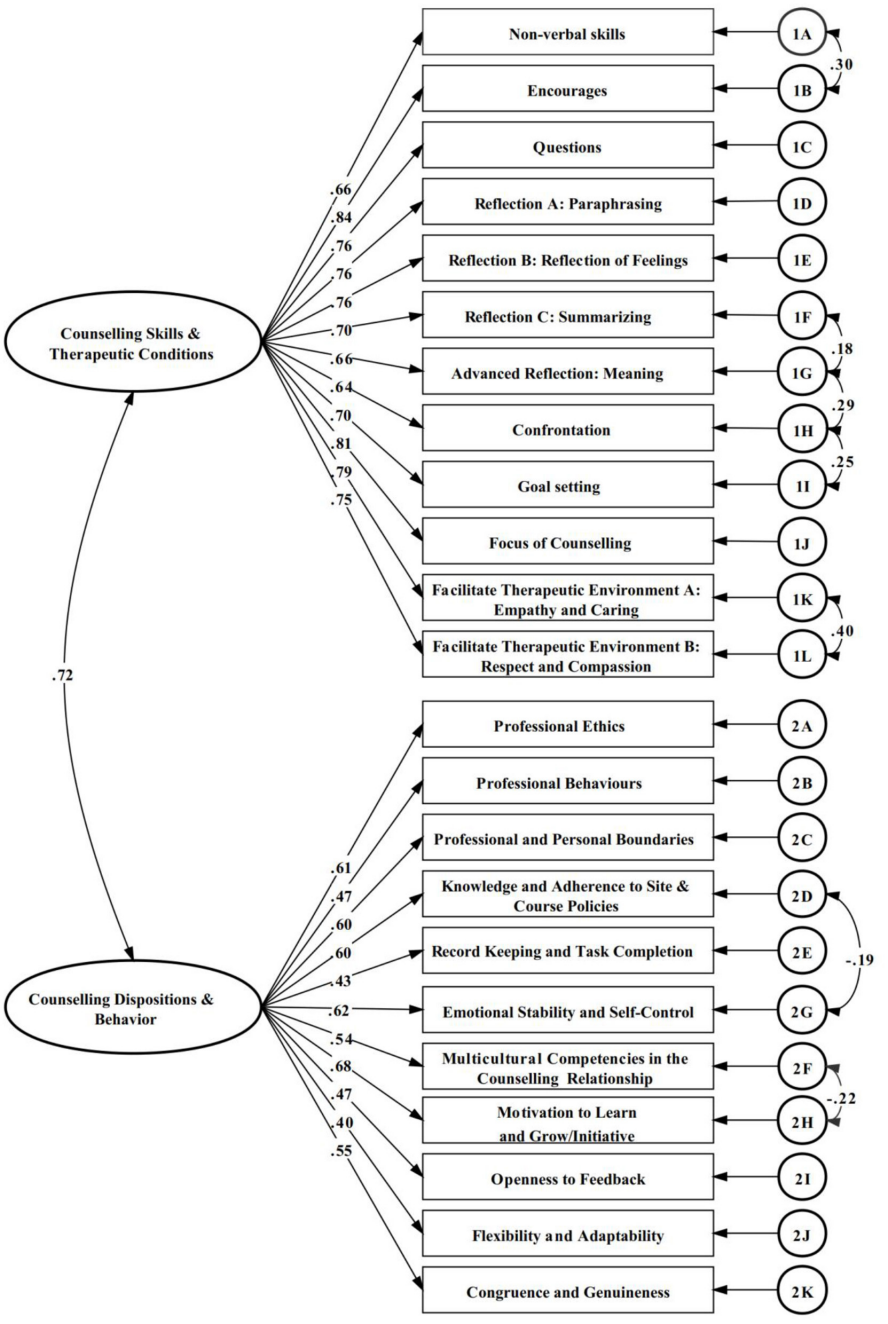
CCS-R modified two-factor model confirmatory analysis results.

The CFA showed that none of the bifactor model, the initial one-factor and two-factor models had a satisfactory fit. The highest modification index was observed for seven pairs of items: (1) 2A: professional ethics/2B: professional behavior, (2) 1F: reflecting summarizing/1G: advanced reflection, (3) 1G: advanced reflection/1H: confronting, (4) 1H: confronting/1I: goal setting, (5) 1K: facilitate therapeutic environmental empathy and caring/1L: facilitate therapeutic environment b: respect and compassion, (6) 2D: knowledge and adherence to site and course policies/2G: emotional stability and self-control and (7) 2F: multicultural competencies in the counseling relationship/2H: motivation to learn and grow/initiative. The pairs of items were modified to have correlated errors. Table 2 summarizes the fit indices of the attempted CFA models. The AIC indicated that the modified two-factor model was the preferable one for the final CCS-R with well performance across all fit indexes: χ^2^/df = 1.26, TLI = 0.95, RMSEA = 0.04, CFI = 0.96, GFI = 0.90, and SRMR = 0.05. All of the factor loadings were >0.32, and no items were removed. The CCS–R items contributing to the counseling skills and therapeutic conditions scale had factor loadings ranging from 0.66 to 0.84 (i.e., good to excellent). Items contributing to the counseling disposition and behavior scale had factor loadings ranging from 0.40 to 0.68 (i.e., fair to very good). In addition, the second-order factor loadings of the counseling skills and therapeutic conditions scale and the counseling disposition and behavior scale to the overall latent factor of counseling competency were excellent, with a value of 0.76. Figure 2 depict the standardized coefficients of the modified two-factor models.

**Table 2:** Fit Indexes from the confirmatory factor analysis with the counseling competencies scale-revised data.

**Model**	**χ^**2**^**	**df**	**χ^**2**^/df**	**AIC**	**TLI**	**RMSEA**	**CFI**	**GFI**	**SRMR**
**One-factor**
Initial	410.24	230	1.78	502.24	0.86	0.06	0.87	0.85	0.06
Modified	321.93	225	1.43	423.93	0.92	0.05	0.93	0.88	0.06
**Two-factor**
Initial	379.01	229	1.66	473.01	0.88	0.06	0.89	0.86	0.06
Modified	279.30	222	1.26	384.50	0.95	0.04	0.96	0.90	0.05
Bifactor	396.41	209	1.90	611.44	0.84	0.07	0.87	0.86	0.08
*Cut-off values*			* <3.0*	–	*≥0.95*	* ≤ 0.06*	*≥0.95*	*≥0.90*	* ≤ 0.08*

*AIC, Akaike Information Criterion; TLI, Tucker–Lewis Index; RMSEA, Root Mean Square Error of Approximation; CFI, Comparative Fit Index; GFI, Goodness-of-fit Index; SRMR, Standardized Root Mean Square Residual*.

**Figure 3:**
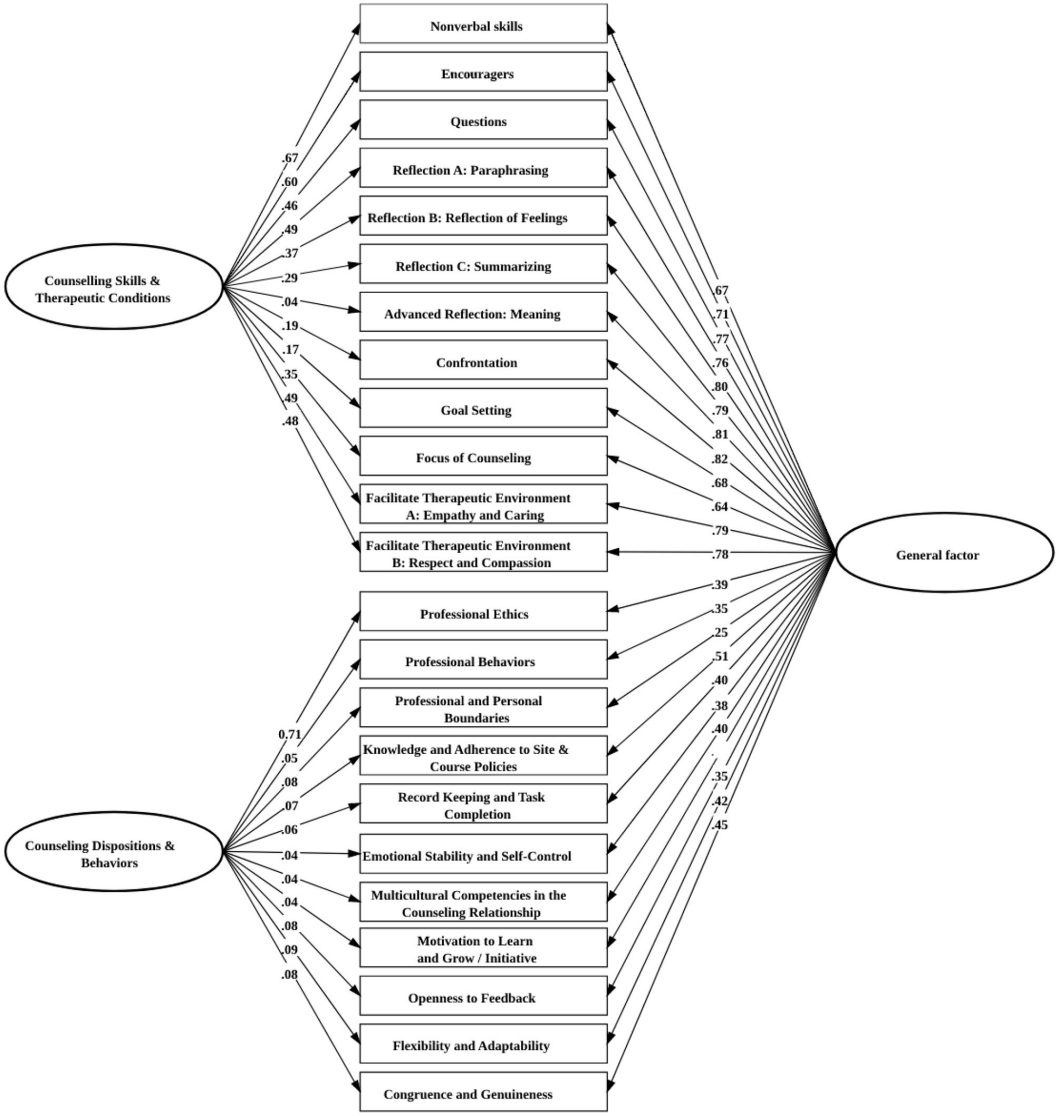
CCS-R bifactor model confirmatory analysis results.

Even not performing best, the bifactor model provided extra information on the Chinese CCS-R. Table 3 displays further statistical indices derived from the bifactor model. The coefficient omega for all scales was found to be 0.95 (>0.70). The omega hierarchical for the global scale was 0.84, and 0.06–0.20 for subscales. Only the global scale had FD >0.9, and *H*-values >0.8, indicating adequate construct replicability Figure 3 depict the standardized coefficients of the bifactor models.

**Table 3:** Bifactor model statistical indices for the CSS-R.

**Scales**	**Omega**	**Omega *H***	**FD**	***H***
Overall counseling competencies scale score	0.95	0.84	0.95	0.95
Counseling skills and therapeutic conditions	0.97	0.20	0.87	0.75
Counseling dispositions and behaviors	0.72	0.06	0.77	0.52

*omega H, omega hierarchical; FD, factor determinacy; H, H index values*.

### Reliability

A descriptive statistical analysis was performed to evaluate the participants' counseling performance (Table 4). The corrected item-scale correlation values ranged between 0.55 and 0.81.The total CCS-R score received a McDonald's omega of 0.876. McDonald's omega values of 0.915 and 0.812 were calculated for factor 1 (Counseling skills and therapeutic conditions), and factor 2 (Counseling disposition and behavior), respectively.

**Table 4:** Descriptive and reliability statistics of the counseling competencies scale-revised.

	**Mean (SD)**	Item total correlation^*^
**Counseling skills and therapeutic conditions McDonald's omega 0.915**		
Item 1A	4.02 (0.66)	0.68
Item 1B	3.96 (0.72)	0.76
Item 1C	3.84 (0.73)	0.77
Item 1D	3.80 (0.71)	0.76
Item 1E	3.56 (0.76)	0.77
Item 1F	3.62 (0.71)	0.74
Item 1G	3.63 (0.72)	0.72
Item 1H	3.35 (0.71)	0.72
Item 1I	3.67 (0.78)	0.59
Item 1J	3.86 (0.59)	0.58
Item 1K	3.82 (0.78)	0.81
Item 1L	3.82 (0.70)	0.80
**Counseling dispositions and behaviors McDonald's omega 0.812**		
Item 2A	3.82 (0.57)	0.79
Item 2B	3.86 (0.71)	0.65
Item 2C	3.82 (0.69)	0.68
Item 2D	3.94 (0.53)	0.58
Item 2E	3.94 (0.39)	0.66
Item 2F	3.71 (0.76)	0.64
Item 2G	3.75 (0.71)	0.70
Item 2H	3.88 (0.53)	0.78
Item 2I	3.77 (0.41)	0.55
Item 2J	3.62 (0.75)	0.71
Item 2K	3.79 (0.66)	0.56
**Overall counseling competencies scale-revised McDonald's omega 0.876**		

* *A correlation between the question score and the overall assessment score*.

As shown in Table 5, the ICC value for the test-retest reliability of the overall scale was 0.63 (*p* < 0.001), and the values for individual items ranged from 0.42 to 0.76 for each items, indicating that the tool was acceptably stable. The ICC values for factor 1 and factor 2 were 0.73 and 0.51, indicating good and acceptable stability, respectively.

**Table 5:** Test-retest reliability and interater reliability of the counseling competencies scale-revised.

	**Test-retest reliability ICC (95% CI) (*n* = 53)**	**Inter-rater reliability ICC (95% CI) (*n* = 208)**
**Counseling skills and therapeutic conditions**	*0.73 (0.52, 0.84)*	*0.93(0.89, 0.95)*
Item 1A	0.58 (0.27, 0.75)	0.82 (0.54, 0.91)
Item 1B	0.59 (0.30, 0.77)	0.82 (0.55, 0.91)
Item 1C	0.66 (0.40, 0.80)	0.83 (0.58, 0.92)
Item 1D	0.56 (0.25, 0.75)	0.76 (0.48, 0.87)
Item 1E	0.73 (0.36, 0.89)	0.80 (0.51, 0.90)
Item 1F	0.41 (0.03, 0.65)	0.79 (0.52, 0.89)
Item 1G	0.50 (0.15, 0.71)	0.75 (0.22, 0.89)
Item 1H	0.58 (0.44, 0.69)	0.79 (0.52, 0.89)
Item 1I	0.42 (0.09, 0.66)	0.72 (0.16, 0.87)
Item 1J	0.44 (0.02, 0.68)	0.73 (0.48, 0.85)
Item 1K	0.56 (0.24, 0.75)	0.81 (0.51, 0.90)
Item 1L	0.50 (0.12, 0.71)	0.71 (0.25, 0.86)
**Counseling dispositions and behaviors**	*0.51 (0.34, 0.68)*	*0.62 (0.44, 0.75)*
Item 2A	0.51 (0.34, 0.73)	0.43 (0.08, 0.51)
Item 2B	0.69 (0.34, 0.84)	0.67 (0.46, 0.81)
Item 2C	0.47 (0.25, 0.57)	0.59 (0.32, 0.73)
Item 2D	0.76 (0.38, 0.91)	0.43 (0.12, 0.53)
Item 2E	0.76 (0.38, 0.91)	0.39 (0.18, 0.51)
Item 2F	0.49 (0.40, 0.54)	0.76 (0.30, 0.89)
Item 2G	0.54 (0.37, 0.73)	0.73 (0.42, 0.85)
Item 2H	0.79 (0.53, 0.96)	0.52(0.30, 0.67)
Item 2I	0.48 (0.34, 0.51)	0.59 (0.44, 0.71)
Item 2J	0.65 (0.32, 0.85)	0.76 (0.22, 0.89)
Item 2K	0.63 (0.39, 0.85)	0.68 (0.43, 0.81)
**Overall scores**	*0.63 (0.34, 0.79)*	*0.90 (0.83, 0.94)*

*ICC, intraclass correlation coefficient; CI, confidence interval*.

The ICC for the IRR of the overall scale was 0.90 (*p* < 0.001), indicating a high degree of agreement between the counseling supervisors. The ICC value for factor 1 was 0.93, indicating a high degree of agreement between the supervisors and the assignment of similar scores for the participants' counseling skills and therapeutic conditions across the raters. The IRR value of 0.62 for factor two indicated that the counseling supervisors had reached a moderate degree of agreement and suggested that the participants' counseling dispositions and behaviors were scored less similarly across the raters.

### Validity

Significant positive correlations were observed between the total CCS-R score and scores of knowledge on drug abuse (Pearson's γ = 0.833, *p* = 0.015) indicating a large convergent validity, and between the total CCS-R score performance checklist (Pearson's γ = 0.781, *p* < 0.001), indicating a moderate concurrent validity.

## Discussion

This is the first study to rigorously translate the CCS-R into Chinese and evaluate its psychometric properties by evaluating a cohort of counselors-in-training in China. The analysis revealed satisfactory reliability and validity. The study results support the use of this tool to measure the counseling competencies of trainees in the Chinese population.

The CFA conducted in this study showed that the modified two-factor model was the best fitting one, which supported the two-factor structure of the CCS-R. The factor loadings were >0.70, with good fit indexes. The information derived from the bifactor analysis demonstrated the essential unidimensionality of the instrument. The overall score scale had a high omega *H* of 0.84, which is merely 11% lower than its omega, whereas the subscales had a generally low omega *H*. Second, only the overall score had adequate FD and *H* values, indicating that the overall score has adequate factor determinacy and construct replicability. The results support the construct validity of the Chinese CCS-R and the use of this scale to measure the competencies of Chinese counselors-in-training with respect to skills, therapeutic conditions, disposition, and behavior. However, in the modified two-factor model, there were seven pairs of error covariance, which also appeared in the original scale, a modified version of scale should be developed in the future.

The corrected item-scale correlation is used to assess the extent to which an item is associated with its corresponding scale, and a value >0.3 must be achieved (Tabachnick and Fidell, 2013). In this study, the corrected item-scale correlation values ranged between 0.55 and 0.81. Moreover, the Chinese CCS-R had satisfactory internal reliability, with overall McDonald's omega values >0.80. The ICC value for the test-retest reliability of the overall scale was 0.63 (*p* < 0.001), indicating that the tool had acceptable stability. According to a previous study, brief feedback from supervisors was reported to promote the skills of counselors (Muñoz et al., 2019). Therefore, the observed test-retest discrepancies may be due to the feedback and suggestions given to the counselor-in-training at the end of performance evaluation, as this may have led to improvements in the students' counseling skills in consequent performances. This possibility was supported by the observation that the mean score of the subsequent performance (mean = 89.2, SD = 7.7) was higher than that of the first performance (mean = 86.0, SD = 8.2).

The results of the IRR analyses of the CCS-R domains and total scale (counseling skills and therapeutic conditions,0.93; counseling dispositions and behaviors,0.62; total CCS–R,0.90) were acceptable. Additional training in the use of the CCS–R might improve the CCS–R IRR scores in the counseling disposition and behavior domain. Still, the raters reported that the items in factor 2 were difficult to evaluate because they required long-term observation. In future studies, the items in factor 2 could be modified for a better evaluation.

The associations of the Chinese CCS-R with the performance checklist (Pearson's γ = 0.781, *p* < 0.001) and score of knowledge on drug abuse (Pearson's γ = 0.833, *p* = 0.015) were confirmed. These large and moderate correlations reflected acceptable concurrent and construct validity, respectively. The findings were consistent with those of previous studies that identified associations between the quality of the counseling service and knowledge and essential questions [Hensley et al., 2003; Association for Counselor Education and Supervision (ACES), 2011]. The convergent validity of the Chinese CCS-R demonstrated the hypothesized associations that counselors with higher scores on the knowledge on drug abuse had focused on counseling knowledge, and that completing comprehensive counseling procedures could enable better counseling competencies.

### Limitation

This study had several limitations. First, the sample population was restricted to a single addiction counselor training program, and therefore, the results might not be representative of all counselor preparation programs. Future studies should involve multiple centers and different counseling settings to ensure the generalizability of the findings. In addition, the relatively small sample size of 208, compared to the sample of more than 1,000 participants in the original study for the scale development, may have limited the CFA. A study with a larger population is required to further assess the validity of the Chinese CCS-R and establish the norms that will facilitate the interpretation of individual results. Second, although the IRR ICC scores for the total CCS–R (0.90) and CCS–R Factor 1 (0.93) were high, the lower IRR ICC score for CCS–R Factor 2 (0.62) warrants consideration. Third, considering this study was conducted attaching to a counseling training programme. Therefore, we did not control the feedback given by the raters after the oral test, which may lead to the observed test-retest discrepancies. In addition, due to the man power limitation, all the raters were female. Whether the all-female rates may influence the rating of the scale during validation was unclear. A study should be conducted with strict control and male raters to further confirm the rest-retest reliability of CCS-R and potential effect. Finally, we assessed the participants' counseling disposition and behavior within a short time, whereas the CCS-R manual suggested an observation period of ~1 semester. In addition, providing the relationship of counseling performance measured using CCS-R to counseling process and outcome could help to enhance the validity of the scale. Due to that long-term observation is needed to recruit sufficient clients and obtain counseling outcomes, this study could not provide such information at current stage. A longer observation and evaluation of the participants' counseling disposition and behavior should be conducted to provide further evidence.

### Implication

According to our literature review, there is a gap in knowledge about the standard counseling training and evaluation provided in China (Zhang, 2018). This study supports the use of the CCS-R to measure the counseling competencies of counselors-in-training in China. The CCS-R provides counseling educators and supervisors with a tool to evaluate trainees' competencies and facilitate discussions about their strengths and areas for growth (Swank et al., 2012; Swank, 2014). Given the above-stated limitations, however, further studies of multiple counseling preparation programs and more diverse samples of counselors-in-training should be conducted to confirm the generalizability of the CCS-R for the overall Chinese population. In addition, studies to explore additional strategies for increasing IRR in the counseling disposition and behavior subscale are warranted. Finally, an examination of the relationship between counselors' CCS–R scores and their clients' outcomes is needed to test the inference that a higher degree of counseling competency can predict increased changes in clients (i.e., criterion-related validity).

## Conclusion

This study addressed a gap in the literature and practice by developing a Chinese-translated version of the CCS-R and examining its psychometric properties. The results suggest the validity and reliability of the Chinese CCS-R for assessing the counseling competencies of counselors-in-training in China. The Chinese CCS-R provides Chinese counseling educators and supervisors with an empirically tested measure and will enable them to evaluate counselors-in-training in a thorough manner and provide formative and summative feedback. These advances will support the further development of counseling training and the promotion of quality counseling services in China.

## Data Availability Statement

The raw data supporting the conclusions of this article will be made available by the authors on reasonable request.

## Ethics Statement

The studies involving human participants were reviewed and approved by Institutional Review Board of the University of Hong Kong/Hospital Authority Hong Kong West Cluster. The patients/participants provided their written informed consent to participate in this study.

## Author Contributions

WX and WHCL designed the study. WX, YL, TL, WHCL, and AC conducted the translation, data collection, and project administration. WX and PS performed the statistical analysis. WX and WHCL wrote the first draft of the manuscript. All authors contributed to and have approved the final manuscript.

## Conflict of Interest

The authors declare that the research was conducted in the absence of any commercial or financial relationships that could be construed as a potential conflict of interest.

## References

[B1] Agarwal R. Rewari B. B. Allam R. R. Chava N. Rathore A. S. (2019). Quality and effectiveness of counselling at antiretroviral therapy centres in India: capturing counsellor and beneficiary perspectives. Int. Health 11, 480–486. 10.1093/inthealth/ihy100 30726940

[B2] American Counseling Association (ACA). (2014). Code of Ethics. Alexandria, VA: American Counseling Association (ACA).

[B3] Association for Counselor Education Supervision (ACES). (2011). Best Practices in Clinical Supervision. Retrieved May 24, 2021, from https://acesonline.net/wp-content/uploads/2018/11/ACES-Best-Practices-in-Clinical-Supervision-2011.pdf

[B4] Beat Drugs Fund (2021). Briefing Note for Outcome Evaluation: Recommended Practice for Questionnaire Administration. Retrieved May 24, 2021, from https://www.nd.gov.hk/en/beat_questions_2010R2.htm

[B5] Bernard J. M. Goodsource R. K. (2013). Fundamentals of Clinical Supervision (5th ed.). Upper Saddle River, NJ: Pearson.

[B6] Bower P. Rowland N. Hardy R. (2003). The clinical effectiveness of counselling in primary care: a systematic review and meta-analysis. Psychol. Med. 33, 203–215. 10.1017/S0033291702006979 12622300

[B7] Brown T. A. (2006). Confirmatory Factor Analysis for Applied Research. New York, NY: Guilford.

[B8] Carroll-Alfano M. A. (2019). Education, counseling, support groups, and provider knowledge of total laryngectomy: the patient's perspective. J. Commun. Disord. 82:105938. 10.1016/j.jcomdis.2019.105938 31557689

[B9] Cicchetti D. V. (1994). Guidelines, criteria, and rules of thumb for evaluating normed and standardized assessment instruments in psychology. Psychol. Assess. 6, 284–290 10.1037/1040-3590.6.4.284

[B10] Council for Accreditation of Counseling Related Educational Programs (CACREP) (2016). 2016 CACREP Standards. Retrieved May 24, 2021, from http://www.cacrep.org/wp-content/uploads/2017/08/2016-Standards-with-citations.pdf

[B11] Dueber D. M. (2017). Bifactor Indices Calculator: A Microsoft Excel-Based Tool to Calculate Various Indices Relevant to bifactor CFA Models. 10.13023/edp.tool.01

[B12] Fiore C. Jaén C. R. Baker T. B. Bailey W. C. Curry S. J. Dorfman S. F. . (2008). Treating Tobacco, Use, and Dependence: 2008 Update. Clinical Practice Guideline. Rockville, MD: US Department of Health and Human Services. Public Health Service.

[B13] Fouad N. Grus C. L. Hatcher R. L. Kaslow N. J. Hutchings P. S. Madson M. B.. (2009). Competency benchmarks: a model for understanding and measuring competence in professional psychology across training levels. Train. Educ. Profess. Psychol. 3, S5–S26 10.1037/a0015832

[B14] Gladding S. T. (2004). Counseling: A Comprehensive Profession (5th ed.). Upper Saddle River, NJ: Merrill/Prentice Hall, 6–7.

[B15] Gorusch R. L. (1983). Factor Analysis. 2nd ed. Hillsdale, NJ: Lawrence Erlbaum Associates.

[B16] Gu H. L. Wen Z. L. Fan X. T. (2017). Structural validity of the Machiavellian Personality Scale: a bifactor exploratory structural equation modeling approach. Pers. Individ. Dif. 105, 116–123. 10.1016/j.paid.2016.09.042

[B17] Hatcher R. L. Lassiter K. D. (2007). Initial training in professional psychology: the practicum competencies outline. Train. Educ. Profess. Psychol. 1, 49–63. 10.1037/1931-3918.1.1.49

[B18] Hensley L. G. Smith S. L. Thompson R. (2003). Assessing competencies of counselors in training: complexities in evaluating personal and professional development. Counselor Educ. Superv. 42, 219–230. 10.1002/j.1556-6978.2003.tb01813.x

[B19] Jackson P. R. Wall T. D. Martin R. Davids K. (1993). New measures of job control, cognitive demand, and production responsibility. J. Appl. Psychol. 78, 753–762. 10.1037/0021-9010.78.5.753

[B20] Karpenko V. Gidycz C. A. (2012). The supervisory relationship and the process of evaluation: recommendations for supervisors. Clin. Superv. 31, 138–158. 10.1080/07325223.2013.730014

[B21] Lambie G. W. Mullen P. R. Swank J. M. Blount A. (2018). The counseling competencies scale: validation and refinement. Measure. Eval. Counsel. Dev. 51, 1–15. 10.1080/07481756.2017.1358964

[B22] Lambie G. W. Swank J. M. (2016). Counseling Competencies Scale–Revised (CCS–R): Training Manual. Orlando, FL: Unpublished manuscript, Department of Child, Family, and Community Sciences, University of Central Florida.

[B23] Lancaster T. Stead L. F. (2005). Individual behavioural counselling for smoking cessation. Cochrane Database Syst. Rev. 3:CD001292. 10.1002/14651858.CD001292.pub2 15846616

[B24] Li W. Chan S. Wang M. P. Ho K. Y. Cheung Y. Chan V.. (2017). An Evaluation of the youth quitline service Young Hong Kong smokers. J. Adoles. Health 60, 584–591. 10.1016/j.jadohealth.2016.11.022 28111012

[B25] McAdams C. R. III. Foster V. A. (2007). A guide to just and fair remediation of counseling students with professional performance deficiencies. Counselor Educ. Superv. 47, 2–13 10.1002/j.1556-6978.2007.tb00034.x

[B26] McFadden A. Siebelt L. Marshall J. L. Gavine A. Girard L. C. Symon A.. (2019). Counselling interventions to enable women to initiate and continue breastfeeding: a systematic review and meta-analysis. Int. Breastfeed. J. 14:42. 10.1186/s13006-019-0235-8 31649743 PMC6805348

[B27] Muñoz K. Ong C. W. Whicker J. Twohig M. (2019). Promoting counseling skills in audiology clinical supervisors: considerations for professional development. Am. J. Audiol. 28, 1052–1058. 10.1044/2019_AJA-19-0060 31644313

[B28] Mvududu N. H. Sink C. A. (2013). Factor analysis in counseling research and practice. Counsel. Outcome Res. Eval. 4, 75–98. 10.1177/2150137813494766

[B29] Ng R. M. Lee C. K. Liu J. Luo J. Zu S. Mi S.. (2017). Psychotherapy services in China: current provisions and future development. J. Contemp. Psychother. 47, 87–94. 10.1007/s10879-016-9345-4

[B30] Norwood S. L. (2000). Research Strategies for Advanced Practice Nurses (No. 895). Prentice Hall.

[B31] Nunnally J. C. (1994). Psychometric Theory (3rd ed.). Tata McGraw-hill education.

[B32] Ohrbach R. Bjorner J. Jezewski M. John M. Lobbezoo F. (2013). Guidelines for Establishing Culture Equivalency of Instruments. Committee for Translation and Protocols International RDC/TMD Consortium Network. Retrieved May 24, 2021, from https://ubwp.buffalo.edu/rdc-tmdinternational/wp-content/uploads/sites/58/2017/01/Guidelines-for-Translation-and-Cultural-Equivalency-of-Instruments-2013_05_118608.pdf

[B33] Rodriguez A. Reise S. P. Haviland M. G. (2016). Applying bifactor statistical indices in the evaluation of psychological measures. J. Pers. Assess. 98, 223–237. 10.1080/00223891.2015.1089249 26514921

[B34] Shultz K. S. Whitney D. J. Zickar M. J. (2020). Measurement Theory in Action: Case Studies and Exercises. Routledge. 10.4324/9781003127536

[B35] Swank J. M. (2014). Counseling competencies: a comparison of supervisors' ratings and student supervisees' self-ratings. Counsel. Outcome Res. Eval. 5, 17–27. 10.1177/2150137814529147

[B36] Swank J. M. Lambie G. W. (2012). The assessment of CACREP core curricular areas and students learning outcomes using the Counseling Competencies Scale. Counsel. Outcomes Res. Eval. 3, 116–127. 10.1177/2150137812452560

[B37] Swank J. M. Lambie G. W. Witta E. L. (2012). The counseling competencies scale: a measure of counseling skills, dispositions, and behaviors. Counselor Educ. Superv. 51, 189–206. 10.1002/j.1556-6978.2012.00014.x

[B38] Tabachnick B. G. Fidell L. S. (2013). Using Multivariate Statistics (6th ed.). Boston, MA: Allyn and Bacon.

[B39] Tsang S. Royse C. F. Terkawi A. S. (2017). Guidelines for developing, translating, and validating a questionnaire in perioperative and pain medicine. Saudi J. Anaesthesia 11, S80–S89. 10.4103/sja.SJA_203_17 28616007 PMC5463570

[B40] Wynd C. A. Schmidt B. Schaefer M. A. (2003). Two quantitative approaches for estimating content validity. West. J. Nurs. Res. 25, 508–518. 10.1177/0193945903252998 12955968

[B41] Zhang L. (2018). Cultivating the therapeutic self in China. Med. Anthropol. 37, 45–58. 10.1080/01459740.2017.1317769 28402134

[B42] Zhao X. (2014). Opportunities and challenges for promoting psychotherapy in contemporary China. Shanghai. Arch. Psychiatry 26, 157–159. 10.3969/j.issn.1002-0829.2014.03.007 25114491 PMC4118013

