# Supplementary material for: Adaptation and Psychometric Evaluation of the Chinese Counseling Competencies Scale-Revised
Source: Front Psychol. 2021 Jun 21;12:688539. doi: 10.3389/fpsyg.2021.688539 (PMC8255382; doi:10.3389/fpsyg.2021.688539)
Supplement: Supplementary file 1 [file Table_1.DOCX]

Supplementary Table 1.1 The content validity score of the relevance of the translated Counseling Competencies Scale-Revised

| Items | Expert 1 | Expert 2 | Expert 3 | Expert 4 | Expert 5 | No. in agreement | I-CVI |
| --- | --- | --- | --- | --- | --- | --- | --- |
| *Part 1: Counseling skills and therapeutic conditions mean S-CVI=0.983* | | | | | | | |
| 1.A | 4 | 4 | 4 | 4 | 4 | 5 | 1 |
| 1.B | 4 | 4 | 3 | 4 | 4 | 5 | 1 |
| 1.C | 4 | 4 | 3 | 4 | 4 | 5 | 1 |
| 1.D | 4 | 4 | 4 | 4 | 4 | 5 | 1 |
| 1.E | 4 | 4 | 3 | 4 | 4 | 5 | 1 |
| 1.F | 4 | 4 | 2 | 3 | 4 | 4 | 0.8 |
| 1.G | 3 | 4 | 3 | 4 | 4 | 5 | 1 |
| 1.H | 3 | 4 | 3 | 4 | 4 | 5 | 1 |
| 1.I | 3 | 4 | 4 | 4 | 4 | 5 | 1 |
| 1.J | 3 | 4 | 4 | 4 | 4 | 5 | 1 |
| 1.K | 4 | 4 | 3 | 4 | 4 | 5 | 1 |
| 1.L | 4 | 4 | 3 | 4 | 4 | 5 | 1 |
| *Part 2: Counseling dispositions and behaviors mean S-CVI=0.855* | | | | | | | |
| 2.A | 2 | 4 | 4 | 2 | 4 | 3 | 0.6 |
| 2.B | 4 | 3 | 3 | 4 | 4 | 5 | 1 |
| 2.C | 3 | 3 | 3 | 4 | 4 | 5 | 1 |
| 2.D | 3 | 2 | 2 | 4 | 4 | 3 | 0.6 |
| 2.E | 4 | 3 | 3 | 4 | 4 | 5 | 1 |
| 2.F | 3 | 4 | 2 | 4 | 4 | 4 | 0.8 |
| 2.G | 4 | 4 | 3 | 2 | 4 | 4 | 0.8 |
| 2.H | 3 | 3 | 2 | 4 | 3 | 4 | 0.8 |
| 2.I | 3 | 3 | 3 | 4 | 4 | 5 | 1 |
| 2.J | 3 | 4 | 4 | 4 | 4 | 5 | 1 |
| 2.K | 3 | 3 | 2 | 4 | 3 | 4 | 0.8 |
| *Overall Counseling Competencies scale-revised* *mean S-CVI=0.922* | | | | | | | |

Note: I-CVI: item content validity index; S-CVI: scale content validity index.

Supplementary table 1.2 The semantic equivalence score of the appropriateness of the translated Counseling Competencies Scale-Revised

| Items | Expert 1 | Expert 2 | Expert 3 | Expert 4 | Expert 5 | No. in agreement | I-CVI |
| --- | --- | --- | --- | --- | --- | --- | --- |
| *Part 1: Counseling skills and therapeutic conditions mean semantic equivalence score =0.917* | | | | | | | |
| 1.A | 4 | 3 | 3 | 4 | 4 | 5 | 1 |
| 1.B | 4 | 3 | 2 | 4 | 3 | 4 | 0.8 |
| 1.C | 3 | 3 | 2 | 4 | 3 | 4 | 0.8 |
| 1.D | 3 | 3 | 2 | 4 | 3 | 4 | 0.8 |
| 1.E | 4 | 4 | 3 | 4 | 3 | 5 | 1 |
| 1.F | 4 | 3 | 3 | 4 | 4 | 5 | 1 |
| 1.G | 4 | 3 | 2 | 4 | 4 | 4 | 0.8 |
| 1.H | 4 | 3 | 3 | 4 | 3 | 5 | 1 |
| 1.I | 4 | 3 | 4 | 4 | 4 | 5 | 1 |
| 1.J | 4 | 3 | 3 | 4 | 4 | 5 | 1 |
| 1.K | 4 | 3 | 3 | 4 | 3 | 5 | 1 |
| 1.L | 4 | 3 | 2 | 4 | 4 | 4 | 0.8 |
| *Part 2: Counseling dispositions and behaviors mean semantic equivalence score = 0.909* | | | | | | | |
| 2.A | 4 | 3 | 3 | 4 | 4 | 5 | 1 |
| 2.B | 4 | 3 | 2 | 4 | 3 | 4 | 0.8 |
| 2.C | 4 | 4 | 2 | 4 | 4 | 4 | 0.8 |
| 2.D | 3 | 3 | 3 | 4 | 4 | 5 | 1 |
| 2.E | 4 | 4 | 3 | 4 | 3 | 5 | 1 |
| 2.F | 4 | 3 | 3 | 4 | 3 | 5 | 1 |
| 2.G | 3 | 3 | 2 | 4 | 3 | 4 | 0.8 |
| 2.H | 3 | 4 | 2 | 4 | 4 | 4 | 0.8 |
| 2.I | 4 | 4 | 3 | 4 | 4 | 5 | 1 |
| 2.J | 4 | 3 | 3 | 4 | 4 | 5 | 1 |
| 2.K | 4 | 3 | 2 | 3 | 3 | 4 | 0.8 |
| *Overall Counseling Competencies scale-revised* *semantic equivalence score = 0.913* | | | | | | | |
